# Deployment of Constellation with Different Inclinations Using the Nodal Precession and Thrust

**DOI:** 10.3390/s24020631

**Published:** 2024-01-19

**Authors:** Shuailong Zhao, Qinyu Zhu, Xuefeng Tao, Yasheng Zhang

**Affiliations:** Graduate School, Space Engineering University, Beijing 101400, China

**Keywords:** constellation deployment, impulse thrust, continuous thrust, inclination boundary

## Abstract

Strategy selection is critical for constellation deployment missions, both in terms of energy consumption and time cost. The different effects of impulse thrust and continuous thrust on orbit elements lead to a different choice of strategy. With impulse thrust, constellation types are differentiated according to high and medium-low inclinations. Constellations with high inclination are deployed using a strategy that controls the inclination. Constellations with medium-low inclination are deployed using a strategy that controls the semi-long axis. With continuous thrust, constellations are classified according to high, medium, and low inclination. High inclination constellations are deployed with a strategy of controlling inclination. Medium inclination constellations are deployed with a strategy that controls the semi-long axis. Low inclination constellations are deployed with a strategy of directly applying continuous thrust.

## 1. Introduction

Satellite constellations are satellite systems consisting of multiple satellites with stable geometrical configurations of space orbits and fixed spatial and temporal relationships between the satellites to fulfil specific space missions, which can give greater play to the role of satellites and expand the forms of satellite applications [1]. It has advantages that are incomparable to those of a single satellite in terms of global coverage, multiplicity, timeliness, and continuity, but at the same time, its construction costs are also incomparable to those of a single satellite, especially at the deployment phase. At present, with the miniaturization of satellites and the enhancement of space maneuver capabilities, the deployment of satellite constellations has gradually become more flexible and diversified, and the existing methods can be divided into two categories: the direct method and the indirect method.

The Direct Method means that satellites are deployed using a direct launch into the target orbit. The satellite is delivered directly into the target orbital plane by a launch vehicle, and the minimum number of launches required is greater than or equal to the number of orbital planes of the constellation, that is, at least one launch is executed per orbital plane [2,3]; this method has been used in many traditional constellations, such as Iridium and Galileo [4]. The huge cost is a key factor determining how successful the plan can be [5]; frequent rocket launches require large capital and time costs, and the traditional constellation deployment method is difficult to apply to large constellations. To address the problem of deploying large or even mega constellations, there is a need to explore new approaches that save both time and energy.

The Indirect Method means that the satellite separates the plane indirectly and then enters the target orbit. A single launch vehicle is used to launch a group of satellites in an initial orbital plane, and subsequently orbital plane separation is achieved as the satellites maneuver to the target plane. The orbital plane separation here mainly refers to the separation of RAAN (Right Ascension of the Ascending Node), and there are two commonly used means at present. One is to apply thrust to change the RAAN, which is to directly change the RAAN by out-of-plane maneuvering to push the satellite into a different orbital plane [6,7,8]. The second is to change the RAAN by controlling the semi-major axis, a process that takes advantage of the different rates of RAAN progression at different semi-major axis due to the non-spherical uptake of the J2 term to achieve an indirect separation of the planes.

The traditional constellation deployment method is difficult to apply to large constellations because of the large capital and time costs associated with frequent rocket launches. To address the problem of deploying large or even mega constellations, it is necessary to explore new approaches that save both time and energy. Therefore, the indirect method is the research focus of this paper. The development of the indirect method has been facilitated by advances in space propulsion technology, such as efficient miniaturized electric thrusters [9,10] and the exploitation of gravitational perturbation. Innovations in methodology and theory reflected in engineering will provide a significant reduction in launch costs. In exceptional cases, a single launch was sufficient to deploy a complete constellation, such as the FORMOSAT-3/COSMIC mission [11]. The mission deployed six satellites in different planes and took about 20 months in total. In 1993, King and Beidleman [12] first patented the use of the Earth’s gravitational perturbation for the deployment of multi-plane satellite constellations. All satellites are first put into a parking orbit. Satellites belonging to the same target orbital plane are placed in a “pallet” and delivered to their target orbits by a carrier. After a certain period, when the RAAN difference between the two orbital planes reaches the desired value due to the Earth’s gravitational perturbation, the second “pallet” is released from the parking orbit, and the process is repeated until all the “pallets” have been deployed. This method was analyzed for a constellation of small satellites in low orbit in [13], where Crisp also considered the effect of atmospheric drag on total mission failure. This method of deploying a multi-plane satellite constellation using RAAN progression is also used in [14]. One satellite changes its semi-major axis while the other satellite maintains its semi-major axis using atmospheric drag compensation with electric thrusters. Eventually a 180° RAAN separation was achieved between the two satellites. Leppinen [15] proposed a similar indirect approach to deploy a constellation of nanosatellites launched on a single carrier rocket to multiple different orbit planes.

Yang et al. [16] proposed a method to deploy a constellation of satellites using a combination of thrust and gravitational perturbation. McGrath et al. used the same strategy of orbit plane separation to solve the deployment problem of circular orbit constellations using a resolved approach [17]. Both Gomez and Yang proposed a combination of thrust and gravitational perturbation for the indirect method of constellation deployment, but the tangential thrust they use is for performing orbital semi-major axis holding of satellites in parked orbits and does not have a direct effect on RAAN separation. Giuseppe [18] simultaneously changes the semi-major axis and inclination to increase the effect of RAAN progression on the target orbit plane, improving the existing deployment method in terms of energy optimality. Zhang Yulin [19], in performing RAAN reconstruction of different orbit planes, similarly noticed that the different inclinations of the orbit planes would lead to different rates of their RAAN progression. Based on the impulse maneuver theory, he found the inclination boundaries for RAAN reconfiguration of different orbital planes: for high inclination orbits, RAAN separation is achieved by using inclination separation; for medium and low inclination orbits, RAAN separation is achieved by using orbital semi-major axis separation. It is necessary to note that, with the development of space electric propulsion, the thrust of electric propulsion can be up to 3 mN–1 N, and the specific impulse can be up to 800–3000 s [20,21]. using continuous thrust to directly change the satellite orbit plane of the RAAN can save a lot of time, but the corresponding energy consumption is pending to be explored.

This paper expands upon the theory of impulse thrust to include continuous thrust and derives an inclination boundary for the efficient separation of RAAN in both thrust modes. It is shown that the velocity increment required to apply continuous thrust to directly change the RAAN is related to inclination. Then, inclination boundary is derived to distinguish between using the method of applying thrust or controlling other orbit elements to achieve RAAN separation. Section 2 derives the relevant mathematical formulas, including the effect of J2 perturbation on orbit elements, the equations of motion under continuous thrust, and the velocity increments required to change different orbit elements for both thrust modes. Section 3 analyzes the deployment strategies under impulse thrust and continuous thrust conditions. Experimental simulations are performed in Section 4 to validate the proposed strategy theory.

## 2. Problem Formulation

### 2.1. Effects of J2 Perturbation

Neglecting the effect of the short-periodic term, the effect of the J2 perturbation [22,23] on the classical orbital elements is as follows.

(1) $$\left\{\begin{array}{l} \frac{da}{dt}=0\\ \frac{de}{dt}=0\\ \frac{di}{dt}=0\\ \frac{d\Omega }{dt}=-\frac{3J_{2}R_{e}^{2}}{2p^{2}}n\cos i\\ \frac{dM}{dt}=\frac{3J_{2}R_{e}^{2}}{2p^{2}}n\left(2-\frac{5}{2}\sin^{2}i\right)\\ \frac{d\omega }{dt}=\frac{3J_{2}R_{e}^{2}}{2p^{2}}n\left(1-\frac{3}{2}\sin^{2}i\right)\sqrt{1-e^{2}} \end{array}\right.$$

where *a* is the semi-major axis, *e* is the eccentricity, *i* is the inclination, Ω is the RAAN, *M* is the mean anomaly, *ω* is the argument of perigee, *n* is the angular velocity, *p* = *a*(1 − *e*^2^) and *p* is the semilatus rectum of the conic curve. Under the J2 perturbation, the semi-major axis, eccentricity, and inclination do not change, and the change in RAAN is related to the semi-major axis, eccentricity, and inclination.

The control of eccentricity is complicated and energy consuming, which is beyond the study scope of this paper. In this paper, we focus on analyzing the influence of semi-major axis and inclination on the variation law of RAAN. Under the non-spherical J2 perturbation of the Earth, the change rate of RAAN is derived from the semi-major axis and inclination as follows.

(2) $$\frac{\partial {\dot{\Omega }}_{J_{2}}}{\partial a}=\frac{\Delta {\dot{\Omega }}_{J_{2}}}{\Delta a}=\frac{21J_{2}R_{e}^{2}}{4ap^{2}}n\cos i$$

(3) $$\frac{\partial {\dot{\Omega }}_{J_{2}}}{\partial i}=\frac{\Delta {\dot{\Omega }}_{J_{2}}}{\Delta i}=\frac{3J_{2}R_{e}^{2}}{2p^{2}}n\sin i$$

For near-circular orbits, there are:

(4) $$\Delta a=\frac{4a^{3}\Delta {\dot{\Omega }}_{J_{2}}}{21J_{2}R_{e}^{2}n\cos i}$$

(5) $$\Delta i=\frac{2a^{2}\Delta {\dot{\Omega }}_{J_{2}}}{3J_{2}R_{e}^{2}n\sin i}$$

### 2.2. Equations of Motion for Continuous Thrust

The orbital equation of motion under continuous thrust is the key theory for constellation deployment. The perturbation equation [24] under continuous thrust is as follows.

(6) $$\left\{\begin{array}{l} \frac{da}{dt}=\frac{2a^{2}}{h}\left\{ef_{R}\sin\nu +\frac{p}{r}f_{C}\right\}\\ \frac{de}{dt}=\frac{1}{h}\left\{pf_{R}\sin\nu +f_{C}\left((p+r)\cos\nu +re\right)\right\}\\ \frac{di}{dt}=\frac{r\cos\vartheta }{h}f_{N}\\ \frac{d\Omega }{dt}=\frac{r\sin\vartheta }{h\sin i}f_{N}\\ \frac{d\omega }{dt}=\frac{1}{he}\left\{-pf_{R}\cos\nu +\left(p+r\right)f_{C}\sin\nu \right\}-\frac{r\sin\vartheta }{h\sin i}f_{N}\cos i \end{array}\right.$$

where *ϑ* = *ν* + *ω*, *ν* is the true proximity angle, *h* is the orbital angular momentum, and *μ* is the Earth’s gravitational parameter. The thrust (*f_R_*, *f_C_*, *f_N_*) applied by the electric thrusters can be expressed in the RCN (Radial Circumferential Normal) reference system. The radial component *f_R_* is in the orbit plane, along the zenith; the normal component *f_N_* is perpendicular to the orbit plane, in the same direction as $\vec{R}\times \vec{V}$; and the circumferential component *f_C_* is in the orbit plane, perpendicular to the radial vector in the orbit plane. 

**Figure 1:**
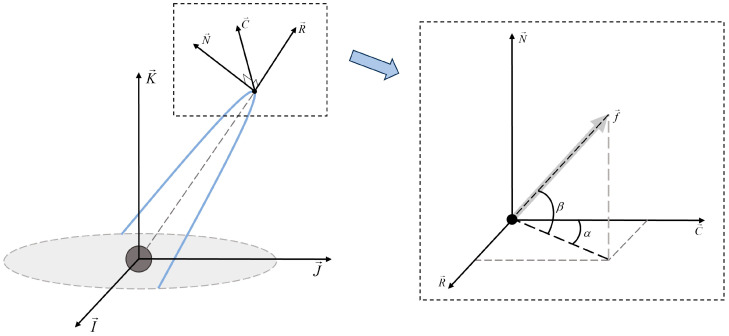
RCN reference system and thrust Angle.

Under the influence of continuous thrust, there is a complex coupling relationship between the orbit elements. As can be seen from Equation (6), the variation of the semi-major axis of the orbit is directly related to the radial component *f_R_* and the circumferential component *f_C_*; the variation of the inclination and the RAAN is directly related to the normal component *f_N_*. The direct correlation with the semi-major axis implies that there is some correlation between inclination and RAAN with both the radial component *f_R_* and the circumferential component *f_C_*. The eccentricity and argument of perigee are also affected by forces in all three directions. As shown in Figure 1, the thrust acceleration vector $\vec{f}$ is used to describe the applied thrust. Introducing an azimuth angle *α* (−π < *α* < π) and a pitch angle *β* (−π/2 < *β* < π/2), the acceleration component is expressed as:

(7) $$\left\{\begin{array}{l} f_{R}\;=\;|\vec{f}|\cos\beta \sin\alpha \\ f_{C}\;=\;|\vec{f}|\cos\beta \cos\alpha \\ f_{N}\;=\;|\vec{f}|\sin\beta \end{array}\right.$$

From this, the equations of motion for the orbit after the introduction of the thrust acceleration vector can be obtained.

(8) $$\left\{\begin{array}{l} \frac{da}{dt}=\frac{2a^{2}}{h}\left|\vec{f}\right|\cos\beta \left\{e\sin\nu \sin\alpha +\frac{p}{r}\cos\alpha \right\}\\ \frac{de}{dt}=\frac{1}{h}\left|\vec{f}\right|\cos\beta \left\{p\sin\nu \sin\alpha +\left((p+r)\cos\nu +re\right)\cos\alpha \right\}\\ \begin{array}{l} \frac{di}{dt}=\left|\vec{f}\right|\frac{r}{h}\cos\vartheta \sin\beta \\ \frac{d\Omega }{dt}=\left|\vec{f}\right|\frac{r}{h}\frac{\sin\vartheta }{\sin i}\sin\beta \end{array}\\ \frac{d\omega }{dt}=\frac{1}{he}\left|\vec{f}\right|\cos\beta \left\{-p\cos\nu \sin\alpha +\left(p+r\right)\sin\nu \sin\alpha \right\}-\frac{r\sin\vartheta \cos i}{h\sin i}\sin\beta \end{array}\right.$$

Equation (8) represents the analytical relationship between the thrust angle (*α*, *β*) and the instantaneous change in each orbit element. Observing Equation (8), it can be observed that if the normal thrust does not change direction during an orbital period, the change in inclination and RAAN is almost 0. To maximize the change in inclination or RAAN, the normal thrust needs to change direction at a phase-symmetric position (*ϑ* = ±π/2 or *ϑ* = 0, π). For near-circular orbits, Bruce [25] and Zhao [26] gives equations for the control of the continuous thrust that changes direction at ϑ = ±π/2 and ϑ = 0, π.


(9)The normal thrust changes sign at ϑ=±π/2dadt = ∣f→∣2avcosβcosαdidt = ∣f→∣2πvsinβdΩdt=0dϑdt=v3μ

(10)The normal thrust changes sign at ϑ=0, π.dadt = ∣f→∣2avcosβcosαdidt=0dΩdt = ∣f→∣2πvsinisinβdϑdt=v3μ

### 2.3. Velocity Increment Required to Change the Orbit Elements

Orbital parameters can be changed using both impulsive and continuous thrusts. King [12] and A. Ruggiero [24] derived the velocity increments required to change different orbit elements using impulsive and continuous thrusts for near-circular orbital conditions.


Velocity increment withimpulse thrust ΔVimpulse(11)ΔVimpulsea=12μa1aΔa(12)ΔVimpulsei=μaΔi(13)ΔVimpulseRAAN=μaΔΩ

Equation (11) represents the velocity increment required to change the semi-major axis by impulse thrust, Equation (12) represents the velocity increment required to change the inclination by impulse thrust, and Equation (13) represents the velocity increment required to change the RAAN deficit by impulse thrust.

**Figure 2:**
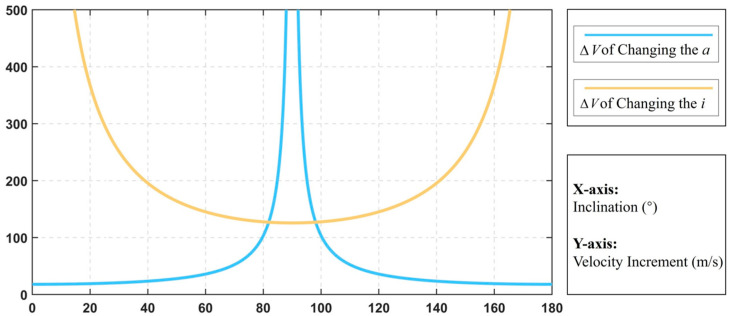
Velocity increment with impulse thrust.

Figure 2 shows the velocity increments required to change the semi-major axis and inclination of the spacecraft at different inclinations under impulse thrust conditions. Observing the solid blue line, it can be found that when the inclination is close to 90° (perpendicular to the equatorial plane), a large velocity increment is required to change the semi-major axis, while a small amount of energy is needed to change the inclination; and observing the solid yellow line, it can be found that when the inclination is close to 0 or 180° (parallel to the equatorial plane), a large velocity increment is required to change the inclination of the orbit, while a small amount of energy is needed to change the semi-major axis of the orbit. The velocity increments required to change the semi-major axis and inclination using impulse thrust have distinct inclination boundaries, and a detailed derivation of the process will be given in the next section. Spacecraft are capable of carrying not only impulse thrust loads but also continuous thrust loads, and the velocity increments required to change the orbital elements under continuous thrust conditions are given below.


Velocity increment withcontinuous thrust ΔVconst(14)ΔVconsta=12μa1aΔa(15)ΔVconsti=π2μaΔi(16)ΔVconstRAAN=π2μasiniΔΩ

Equation (14) represents the velocity increment required to change the semi-major axis for continuous thrust, Equation (15) represents the velocity increment required to change the inclination for continuous thrust, and Equation (16) represents the velocity increment required to change the RAAN deficit for continuous thrust.

**Figure 3:**
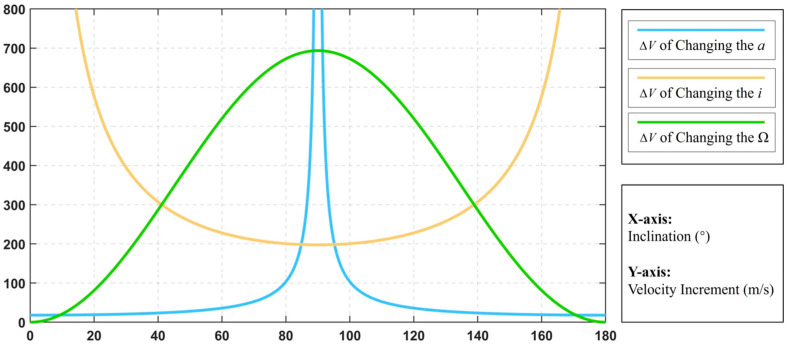
Velocity increment with continuous thrust.

Figure 3 shows the velocity increments required to change the semi-major axis and inclination for the spacecraft at different inclinations under continuous thrust. Comparison of Equations (13) and (16) shows that the velocity increment required to change the RAAN under continuous thrust is also affected by the inclination. As can be seen in Figure 3, there is an intersection of the green solid line with the blue solid line. In other words, when the inclination is small enough, it is more energy efficient to apply thrust directly to control the RAAN.

For changing the semi-major axis of the orbit, the velocity increment required is the same for impulse thrust and continuous thrust. However, for changing the inclination of the orbit, the velocity increment required to use continuous thrust will be π/2 times the velocity increment required to use impulse thrust. In addition, for changing the RAAN, the situation is more complicated, and the velocity increment required to change the RAAN is related to the inclination.

(17) $$\left\{\begin{array}{ll} \Delta V_{const}^{RAAN}<\Delta V_{impulse}^{RAAN} & \;0\leq i<39.5402,\;140.4598<i\leq 180\\ \Delta V_{const}^{RAAN}=\Delta V_{impulse}^{RAAN} & \;i=39.5402,\;i=140.4598\\ \Delta V_{const}^{RAAN}>\Delta V_{impulse}^{RAAN} & \;39.5402<i<140.4598 \end{array}\right.$$

Compared to the velocity increment required to change the orbital inclination, applying continuous thrust to change the RAAN requires an additional term sin(*i*) in the equation, as can also be seen from Equation (4) in Equation (8). Here it is important to note that in terms of the properties of the sinusoidal function, the direct application of thrust to change the RAAN at low inclination will be more fuel and cost efficient than the velocity increments required by the other strategies.

## 3. Multi-Satellite Multiplane Deployment Strategy

### 3.1. Strategy of RAAN Separation

**Figure 4:**
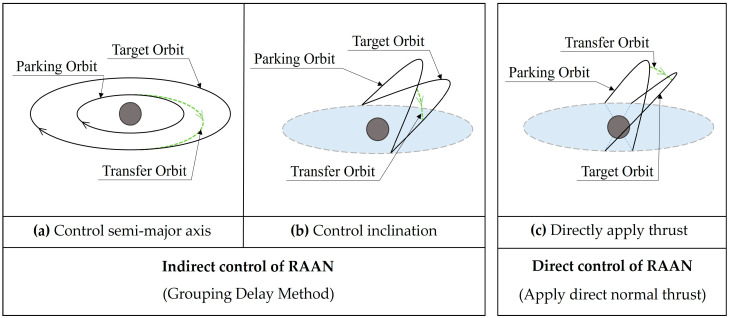
Strategy for RAAN separation.

The simultaneous deployment of multiple satellites in different orbital planes is the main mode of deployment of mega-constellations, and the capability of launching multiple satellites with a single rocket makes this deployment mode feasible. The essence of multi-satellite heterodyne deployment is to realize RAAN separation between multiple satellites, and the selection of an appropriate RAAN separation strategy is the key to constellation deployment. As shown in Figure 4, the RAAN separation strategy is classified into “Indirect control of RAAN” and “Direct control of RAAN”.

**Figure 5:**
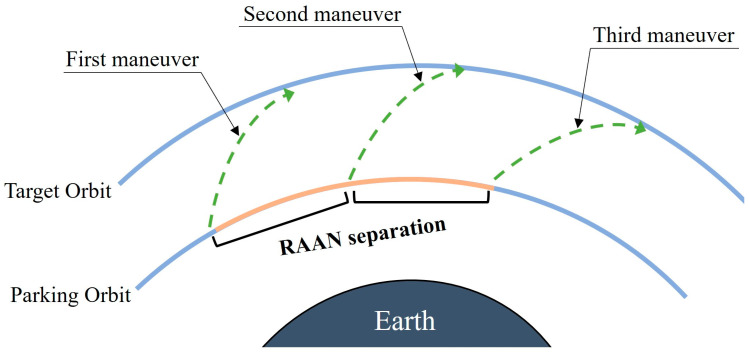
Group delay strategy.

The strategy of “indirect control of RAAN” can be achieved by changing the semi-major axis or inclination as shown in Figure 4a,b. The strategy of “direct control of the RAAN” is to apply a direct normal thrust to the spacecraft, as shown in Figure 4c. Whether impulse or continuous thrust is used, if the method of “indirect RAAN separation by changing orbit elements” is used, the constellation would be deployed using the “group delay” method. The launch vehicle puts all the satellites into a parking orbit, divides all the satellites into three groups, and then conducts the first maneuver, the second maneuver and the third maneuver, with RAAN separations generated during the maneuver time intervals. The schematic of the group delay transfer strategy is shown in Figure 5.

### 3.2. Inclination Boundary with Impulse Thrust

Substitute Equations (4) and (5) into Equations (11) and (12).

(18) $$\Delta V_{impulse}^{a}=\frac{1}{2}\left|\sqrt{\frac{\mu }{a}}\frac{4a^{2}\Delta {\dot{\Omega }}_{J_{2}}}{21J_{2}R_{e}^{2}n\cos i}\right|$$

(19) $$\Delta V_{impulse}^{i}=\left|\sqrt{\frac{\mu }{a}}\frac{2a^{2}\Delta {\dot{\Omega }}_{J_{2}}}{3J_{2}R_{e}^{2}n\sin i}\right|$$

Equations (18) and (19) indicate that an impulse thrust $\Delta V_{impulse}^{a}$ is required to achieve a RAAN drift rate $\Delta {\dot{\Omega }}_{J_{2}}$ by adjusting the semi-major axis, and an impulse thrust $\Delta V_{impulse}^{i}$ is required by adjusting the inclination of the orbit. Comparing the velocity increments required by the two methods, there is

(20) $$\frac{\Delta V_{impulse}^{a}}{\Delta V_{impulse}^{i}}=\left|\frac{\sin i}{7\cos i}\right|$$

**Figure 6:**
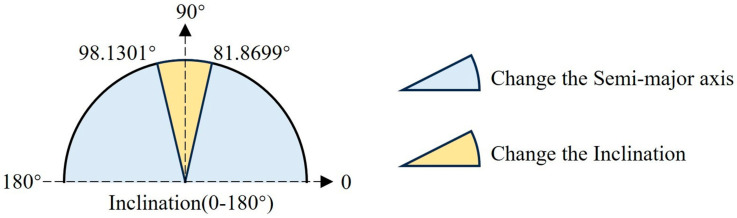
Constellation deployment strategy selection for different inclinations with impulse thrust.

When $\left|\tan i\right|=7$, $\Delta V_{impulse}^{a}/\Delta V_{impulse}^{i}=1$, so *i* = 81.8699° or 98.1301°. As shown in Figure 6, when 0 ≤ *i* < 81.87° or 98.13° < *i* < 180°, adjusting the semi-major axis to separate the orbit planes requires a smaller velocity increment. However, when 81.8699° ≤ i ≤ 98.1301°, it is more fuel efficient to complete the separation of the orbit planes by adjusting the inclination. In practical constellation configurations, the difference between the RAANs of different orbital planes is generally large, and it can be seen from Equation (13) that directly changing the RAAN by applying a normal thrust requires a large velocity increment, so this strategy is not considered here.

### 3.3. Inclination Boundary with Continuous Thrust

Substitute Equations (4) and (5) into Equations (14) and (15).

(21) $$\Delta V_{const}^{a}=\frac{1}{2}\left|\sqrt{\frac{\mu }{a}}\frac{1}{a}\frac{4a^{3}\Delta {\dot{\Omega }}_{J_{2}}}{21J_{2}R_{e}^{2}n\cos i}\right|$$

(22) $$\Delta V_{\mathrm{const}}^{i}=\frac{\pi }{2}\left|\sqrt{\frac{\mu }{a}}\frac{2a^{2}\Delta {\dot{\Omega }}_{J_{2}}}{3J_{2}R_{e}^{2}n\sin i}\right|$$

(23) $$\frac{\Delta V_{\mathrm{const}}^{a}}{\Delta V_{\mathrm{const}}^{i}}=\left|\frac{2\sin i}{7\pi \cos i}\right|$$

Same analytical approach as in Section 3.1, when $\Delta V_{const}^{a}/\Delta V_{const}^{i}=1$, *i* = 84.8035° or 95.1965°. Therefore, when 84.8035° ≤ *i* ≤ 95.1965°, it is more fuel efficient to complete the separation of the orbit planes by adjusting the inclination.

From the fourth equation of Equation (8), it can be seen that the efficiency of changing the RAAN by normal continuous thrust would increase for low inclination. Section 2.3 also analyzes that for low inclination, compared with impulse thrust, the velocity increment required to control the RAAN with continuous thrust will decrease.

According to the characteristics of continuous thrust dynamics [26], the total velocity increment required from *a*_0_ to *a_f_* is $\Delta V_{const}^{a}$.

(24) $$\Delta V_{const}^{a}=\left|\sqrt{\frac{\mu }{a_{0}}}-\sqrt{\frac{\mu }{a_{f}}}\right|$$

where *a*_0_ is the semi-major axis of the parking orbit and *a_f_* is the semi-major axis of the target orbit. Equation (24) is equivalent to Equation (14). Meanwhile, Equation (16) can be expressed as

(25) $$\Delta V_{const}^{RAAN}=\frac{\pi }{2}\left|\sqrt{\frac{\mu }{a_{f}}}\sin i\Delta \Omega \right|$$

**Figure 7:**
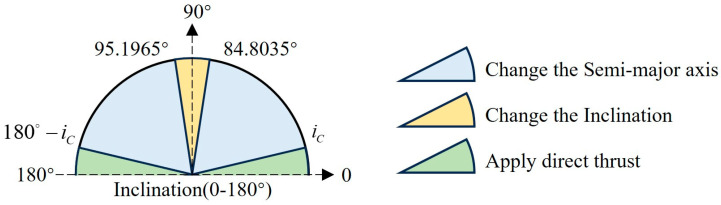
Constellation deployment strategy selection for different inclinations with continuous thrust.

By comparing Equations (24) and (25), we can analyze how to more efficiently change the RAAN under continuous thrust. Observation of the above equation shows that there exists a critical inclination *i_C_* that enables $\Delta V_{const}^{a}/\Delta V_{const}^{RAAN}=1$. As shown in Figure 7, when 0 ≤ *i* < *i_C_* or 180° − *i_C_* < *i* < 180°, a smaller velocity increment is required when thrust is applied directly to achieve RAAN separation. When *i_C_* ≤ *i* < 84.8035° or 95.1965° < *i* < 180° − *i_C_*, fuel savings are better achieved by adjusting the semi-major axis. When 84.8035° ≤ *i* ≤ 95.1965°, adjusting the inclination is more fuel efficient.

The values of *i_C_* in different cases are analyzed below.

**Figure 8:**
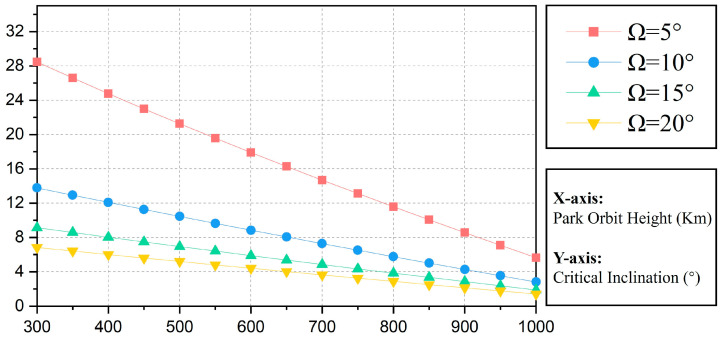
Values of *i_C_* for different scenarios (target orbital height 1200 km).

As can be seen in Figure 8, the smaller the required RAAN separation, the larger the value of *i_C_* is taken; the lower the height of the parking orbit, the smaller the value of *i_C_* is taken.

## 4. Simulation Verification

Theoretical validation needs full-model simulation in real scenarios. The following parameters are set to validate the satellite constellation deployment strategy with two thrust modes (impulse thrust and continuous thrust) proposed in this study.

### 4.1. Multi-Satellite Multiplane Deployment

The space environment simulation parameter settings are shown in Table 1.

The target orbit and satellite thrust parameters are shown in Table 2.

**Figure 9:**
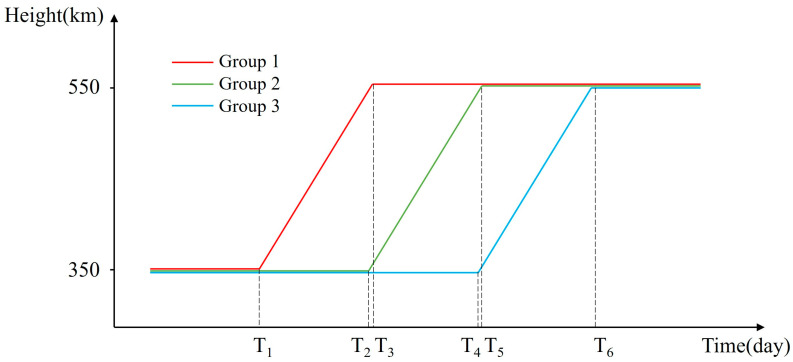
Deployment process.

The following is an example of controlling the semi-major axis (parking orbit height of 350 km) to achieve RAAN separation under continuous thrust condition, to illustrate the indirect control RAAN strategy in detail. The specific operation flow is shown in Table 3, and the process schematic is shown in Figure 9.

As shown in Figure 9, between T1 and T2 is the process of achieving RAAN separation for Group 2 satellites, and between T1 and T4 is the process of achieving RAAN separation for Group 3 satellites. The total velocity increment and time required to deploy the mission at different inclinations are analyzed below. Where the total velocity increment is the sum of the energy required for the three maneuvers and the time required is the total duration of the deployment mission, which is T6.

### 4.2. Low Inclination

**Figure 10:**
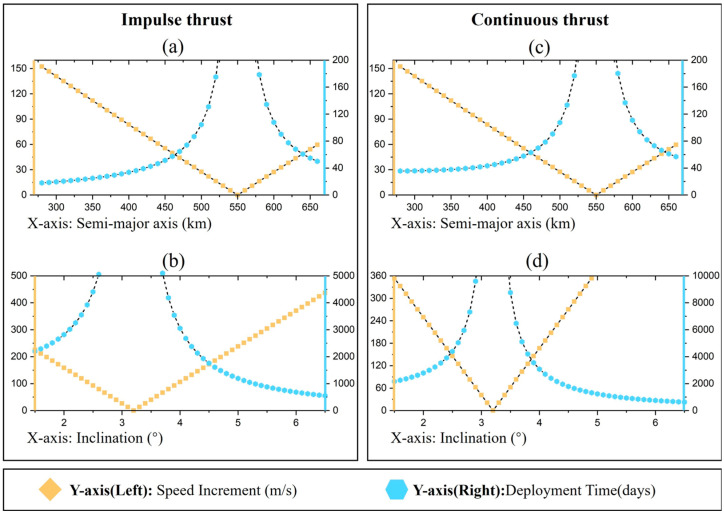
Velocity increments and deployment times required for different thrust modes (*i* = 3.2°). (**a**) Change semi-major axis under impulse thrust; (**b**) Change inclination under impulse thrust; (**c**) Change semi-major axis under continuous thrust; (**d**) Change inclination under continuous thrust.

Figure 10 shows the velocity increment (left Y-axis) and deployment time (right Y-axis) required for constellation deployment at inclination *i* = 3.2°. The energy and time required to control the semi-major axis to indirectly achieve RAAN separation under impulse thrust conditions are indicated in Figure 10a. The horizontal coordinate indicates the altitude of the parking orbit, and the target orbit height is 550 km. When the parking orbit height is 550 km, the velocity increment is 0, and the RAAN separation cannot be achieved, so the required time is infinite. The larger the height difference between the parking orbit and the target orbit, the larger the required velocity increment; but the required deployment time is shorter. Figure 10b represents the energy and time required to indirectly achieve RAAN separation by controlling the inclination under impulse thrust conditions, and the horizontal coordinate represents the orbital inclination of the parking orbit. Similarly, Figure 10c,d represent the energy and time required to control the semi-major axis and inclination under continuous thrust conditions, respectively. The time required for deployment using the controlled orbital semi-major axis under small inclination conditions is much less than the time required to control the inclination for both impulse and continuous thrust.

In this case, applying thrust to directly change the RAAN requires a velocity increment of 1317.85 m/s with impulse thrust and only 115.55 m/s with continuous thrust. Comparing the two scenarios of controlling the semi-major axis and directly applying thrust: (1) when the velocity increment is 115.55 m/s, the semi-major axis of the parking orbit is 343.94 km and the deployment time is 37.41 days; (2) With continuous thrust, the same velocity increment of 115.55 m/s is required for deployment, which takes only 26.74 days, saving 28.51% of the deployment time compared with the former.

### 4.3. Medium Inclination

**Figure 11:**
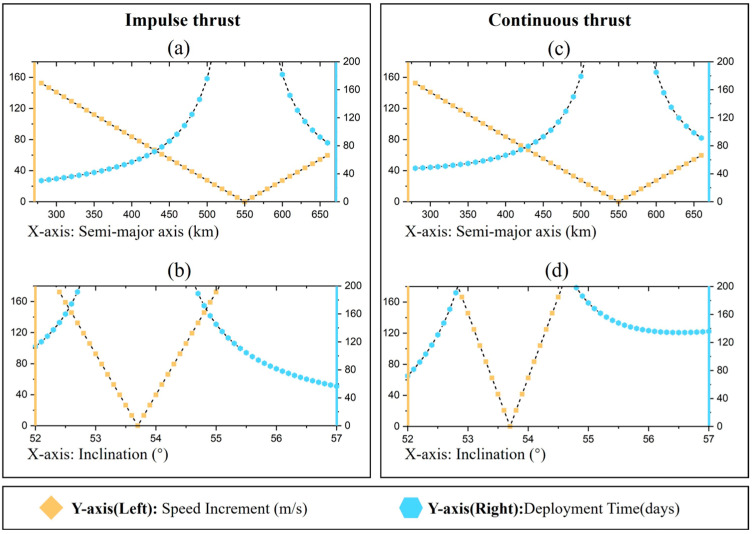
Velocity increments and deployment times required for different thrust modes (*i* = 53.7°). (**a**) Change semi-major axis under impulse thrust; (**b**) Change inclination under impulse thrust; (**c**) Change semi-major axis under continuous thrust; (**d**) Change inclination under continuous thrust.

Figure 11 shows the velocity increment (left Y-axis) and deployment time (right Y-axis) required for constellation deployment with impulse thrust and continuous thrust, respectively, when *i* = 53.7°. From the right Y-axis of the plots in Figure 11, for the medium inclination constellations, the efficiencies of the RAAN changing for controlling the semi-major axis and the inclination are approximated. In this case, the direct application of thrust to change the RAAN requires a velocity increment of 1668.23 m/s and a deployment time of 193.08 days. The strategy of directly applying thrust is not applicable to the deployment of medium inclination constellations, but it may be feasible to simultaneously control the semi-major axis and inclination.

### 4.4. High Inclination

**Figure 12:**
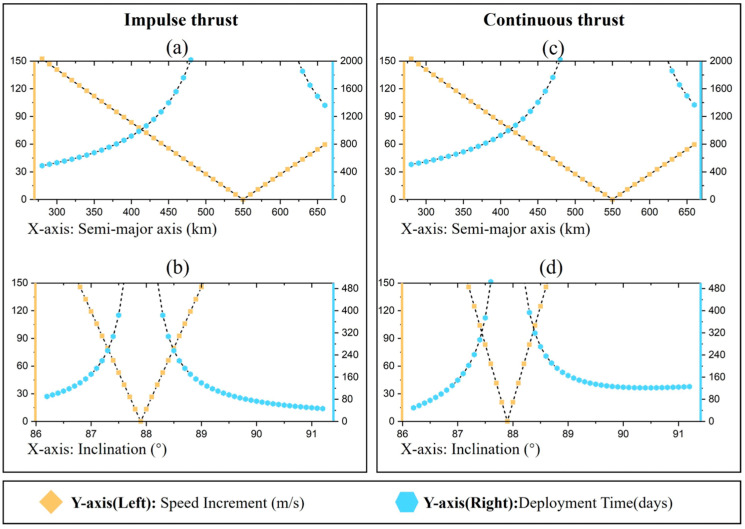
Velocity increments and deployment times required for different thrust modes (*i* = 87.9°). (**a**) Change semi-major axis under impulse thrust; (**b**) Change inclination under impulse thrust; (**c**) Change semi-major axis under continuous thrust; (**d**) Change inclination under continuous thrust.

Figure 12 shows the velocity increment (left Y-axis) and deployment time (right Y-axis) required for constellation deployment with impulse thrust and continuous thrust, respectively, at *i* = 87.9°. From the right Y-axis, for both impulse thrust and continuous thrust, the time required for deploying the constellation with controlled inclination at a small inclination is much smaller than that required for controlling the semi-major axis, indicating that in this case, the deployment of the constellation with controlled inclination is more efficient at high orbital inclination. Similarly, applying direct thrust to change the RAAN of the satellite requires a velocity increment of 2068.64 m/s and takes 239.42 days, which is not applicable to high inclination constellations.

## 5. Conclusions

The study can be applied to the selection of constellation deployment strategies at different inclinations and to the selection of constellation reconfiguration strategies at different inclinations. In the simulations, the proposed method is analyzed and validated and the following conclusions are obtained:

Numbered lists can be added as follows:

1. Due to the effect of integration, the velocity increment required for continuous thrust to change orbit elements is different from the velocity increment required for impulse thrust, and the impact is different for different orbit elements;
2. In the case of impulse thrust, there are orbital inclination boundaries that distinguish between high and medium-low inclinations. For the medium-low inclination constellations, it is more efficient to control the semi-major axis in orbit to achieve RAAN separation; for the high angle constellations, controlling the inclination is more efficient to achieve RAAN separation;
3. In the case of continuous thrust, there exist inclination boundaries distinguishing between high, medium, and low inclinations. For low inclination, it is more efficient to apply thrust directly to change the RAAN. For medium inclination, controlling the semi-major axis to achieve RAAN separation is more efficient. For high inclination, the efficiency of RAAN separation is higher by controlling the inclination;
4. In constellation deployment missions, controlling orbit elements is no longer the only viable option to achieve RAAN separation. The use of continuous thrust to directly change the RAAN at small inclinations saves both time and energy.

Future work may include the optimization of transfer orbits and the derivation of more sophisticated orbital dynamics. The next step to be investigated is the effect of other perturbation forces on the constellation deployment strategy and the use of additional perturbation forces to optimize the deployment strategy.

## Figures and Tables

**Table 1:** Perturbation parameter settings.

Perturbation	Models and Values
Three-body gravity	Sun, Moon and Major Planets: JPL DE405
Tides	Solid Tides: IERS Conventions 2003
Non-spherical gravitational field	Gravity Modelling: EGM2008 Degree: 21; Order: 21
Relativity	IERS Conventions 2003
Solar pressure	Shadow Models: Dual Cone Optical Pressure Coefficient:1.00 Surface-to-Mass Ratio: 0.02 m^2^/kg
Atmospheric drag	Density Model: Jacchia-Roberts Drag coefficient: 2.20 Surface-to-mass ratio: 0.02 m^2^/kg

**Table 2:** Target orbit elements and satellite thrust parameter settings.

Simulation Parameters	Parameter Value
Target orbit height (km)	550
Number of target orbit planes	3
RAAN separation (°)	10
Mass of the satellite (kg)	500
Thruster thrust (mN)	50

**Table 3:** Deployment operation flow.

Step	Operation	Time (Days)
Step 1	After entering the parking orbit, all satellites were divided into three groups, labelled Group 1, Group 2, and Group 3;	T_1_ = 0
Step 2	Group 1 satellites began maneuvering (towards the target orbit);	T_1_ = 0
Step 3	Group 2 satellites began maneuvering (towards the target orbit);	T_2_ = 12.41
Step 4	Group 1 satellites reach the target orbit;	T_3_ = 12.97
Step 5	Group 3 satellites began maneuvering (towards the target orbit);	T_4_ = 24.82
Step 6	Group 2 satellites reach the target orbit;	T_5_ = 25.38
Step 7	Group 3 satellites reached the target orbit and deployment was completed.	T_6_ = 37.79

## Data Availability

Data are contained within the article.
